# PROGNOSTIC FACTORS AND SURVIVAL ANALYSIS IN ESOPHAGEAL CARCINOMA

**DOI:** 10.1590/0102-6720201600030003

**Published:** 2016

**Authors:** Francisco TUSTUMI, Cintia Mayumi Sakurai KIMURA, Flavio Roberto TAKEDA, Rodrigo Hideki UEMA, Rubens Antônio Aissar SALUM, Ulysses RIBEIRO-JUNIOR, Ivan CECCONELLO

**Affiliations:** Cancer Institute of São Paulo State, São Paulo, SP, Brazil

**Keywords:** Esophageal neoplasm, Adenocarcinoma, Squamous cell carcinoma

## Abstract

**Background:**

Despite recent advances in diagnosis and treatment, esophageal cancer still has high mortality. Prognostic factors associated with patient and with disease itself are multiple and poorly explored.

**Aim:**

Assess prognostic variables in esophageal cancer patients.

**Methods:**

Retrospective review of all patients with esophageal cancer in an oncology referral center. They were divided according to histological diagnosis (444 squamous cell carcinoma patients and 105 adenocarcinoma), and their demographic, pathological and clinical characteristics were analyzed and compared to clinical stage and overall survival.

**Results:**

No difference was noted between squamous cell carcinoma and esophageal adenocarcinoma overall survival curves. Squamous cell carcinoma presented 22.8% survival after five years against 20.2% for adenocarcinoma. When considering only patients treated with curative intent resection, after five years squamous cell carcinoma survival rate was 56.6 and adenocarcinoma, 58%. In patients with squamous cell carcinoma, poor differentiation histology and tumor size were associated with worse oncology stage, but this was not evidenced in adenocarcinoma.

**Conclusion:**

Weight loss (kg), BMI variation (kg/m²) and percentage of weight loss are factors that predict worse stage at diagnosis in the squamous cell carcinoma. In adenocarcinoma, these findings were not statistically significant.

## RESUMO

**Racional:**

Apesar dos avanços recentes nos métodos diagnósticos e tratamento, o câncer de esôfago mantém alta mortalidade. Fatores prognósticos associados ao paciente e ao câncer propriamente dito são pouco conhecidos.

**Objetivo:**

Investigar variáveis prognósticas no câncer esofágico.

**Métodos:**

Pacientes diagnosticados entre 2009 e 2012 foram analisados e subdivididos de acordo com tipo histológico (444 carcinomas espinocelulares e 105 adenocarcinomas), e então características demográficas, anatomopatológicas e clínicas foram analisadas.

**Resultados:**

Não houve diferença entre os dois tipos histológicos na sobrevida global. Carcinoma espinocelular apresentou sobrevida de 22,8% em 5 anos, contra 20,2% de adenocarcinoma. Quando considerado somente os tratados com operação com intenção curativa, sobrevida em cinco anos foi de 56,6% para espinocelular e 58% para adenocarcinoma. Para o subtipo espinocelular, tumores pouco diferenciados e extensão tumoral mostraram associação com pior estadiamento oncológico, o que não foi verificado para adenocarcinoma.

**Conclusão:**

Perda de peso, variação de IMC e porcentagem de perda de peso foram fatores associados ao pior estadiamento oncológico para espinocelular, o que não se confirmou para adenocarcinoma.

## INTRODUCTION

Despite recent advances in diagnosis and treatment, esophageal cancer still has high mortality. Mean survival for squamous cell carcinoma (SCC) is 13.95±SD 11.2 months and for esophageal adenocarcinoma (EA) is 13.22±SD 10.23 months^4^ ^,^ ^11^ ^,^ ^13^. 

Prognostic factors associated with patient and with disease itself are multiple and poorly explored. Knowing these parameters can allow a better stratification of high-risk groups^2^ ^,^ ^3^. 

This study aims to assess demographic, clinical and pathological factors in esophageal cancer patients that impact in overall survival and prognostic.

## METHODS

This study retrospectively reviewed esophageal cancer patients that were admitted at an oncology referral center between 2009 and 2012. 

The analyzed variables were age, sex, performance status, past oncologic history, family oncologic history, tumor size, weight loss and body mass index, tumor location, grade of cellular differentiation, oncologic stage, lymphatic dissection, and curative intent resection.

The studied population was composed of 565 individuals (n=565), of which 444 were SCC and 105 EA. The remaining was composed of other less frequent tumors, such as neuroendocrine tumors.

Demographic, pathological and clinical characteristics were analyzed and compared to clinical stage and overall survival at 60 months. Average follow-up was 19.8 months.

### Statistical Analysis

Regarding statistical analysis, to compare group means, ANOVA test was used; to analyze Kaplan-Meier curves, Log-Rank and Wilcoxon tests were used. Influence of prognostic variables was assessed by Cox regression. Significance level admitted was 0.05. 

## RESULTS

**FIGURE 1:**
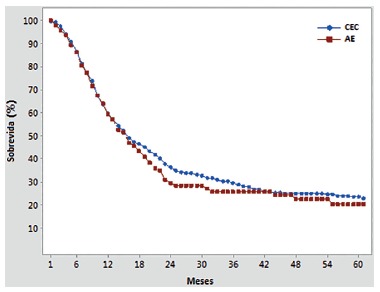
Kaplan-Meier curves of survival, comparing esophageal adenocarcinoma (EA) and squamous cell carcinoma (SCC). There is no statistical difference between the curves (Log-Rank p-value=0.473; Wilcoxon p-value 0.098)

No difference was noted between SCC and EA overall survival curves. After five years, SCC presented 22.81% survival rate against 20.19% for EA (Figure 1)*.*

**FIGURE 2:**
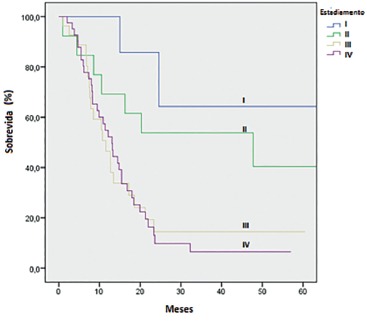
Esophageal adenocarcinoma: overall survival curves according to oncologic stages at diagnosis^3^

**FIGURE 3:**
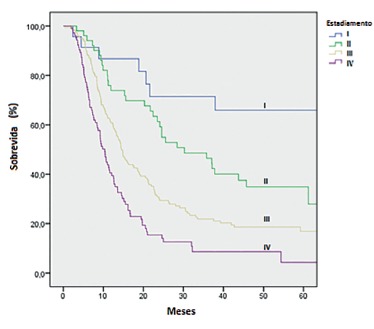
Esophageal squamous cell carcinoma: overall survival curves according to oncologic stages at diagnosis^3^

Of all of the EA patients, 30.4% were eligible for curative intent surgery. This proportion was 20% in SCC patients (p-value for Log-Rank 0.114; for Wilcoxon 0.042). After five years, survival for EA was 58% and for SCC 56.6%. By univariate analysis, curative intention resection was clearly associated to a better survival rate (p-value < 0.001). Figure 2 and 3 present overall survival curves according to oncologic stages at diagnosis.

Longitudinal neoplasm extension at diagnosis was compared to clinical oncologic stage. By Chi-square analysis, it was noted that neoplasm size relate to poor prognosis in SCC (p-value 0.00), but not in EA (p-value 0.173). By univariate Cox regression, only in SCC tumor size was related to survival (p-value 0.001).

Degree of cellular differentiation was related to poor clinical stage in SCC (Chi-Sq=27.831; DF=6; p-value=0.00), but not in EA (Chi-Sq=7.943; DF=6; p-value=0.242).

Weight loss (kg), BMI variation (kg/m²) and percentage of weight loss from initial symptoms to the diagnosis of esophageal carcinoma are factors that predict worse oncologic stage at diagnosis in the SCC. In EA, this finding was not statistically significant (Figure 5). By logistic regression, BMI lower than 20 kg∕m² was a predictor of poor survival rate. 

Considering only patients submitted to curative intent surgery, more than 23 node resection could not reach a statistically significant improvement in survival rate by univariate analysis (p=0.678 in EA and p=0.493 in SCC).

By univariate and multivariate analysis (Tables 1 and 2), variables associated to poor survival rate in EA was weight loss, performance status at the moment of diagnosis and distal location tumors; for SCC, male sex, weight loss, performance status, past history of other malignances and delay in initiating treatment. For both carcinoma types, curative intention resection was more often associated to better prognosis.

**TABLE 1:**
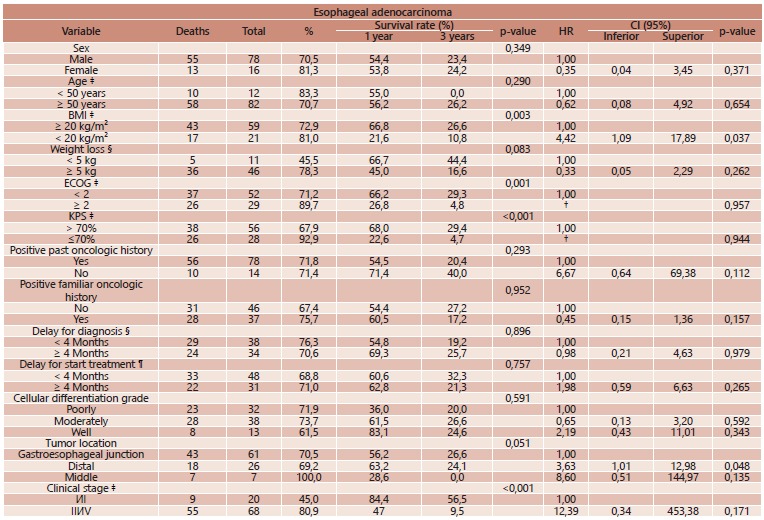
Univariate and multivariate prognostic factors analysis for esophageal adenocarcinoma

†=not possible to estimate; ‡: =at the time of diagnosis; §= time between initial symptoms to diagnosis; ¶=time between diagnosis and initial oncologic treatment; HR=hazard ratio; ECOG=Eastern Cooperative Oncology Group performance status; KPS=Karnofsky performance status

**TABLE 2:**
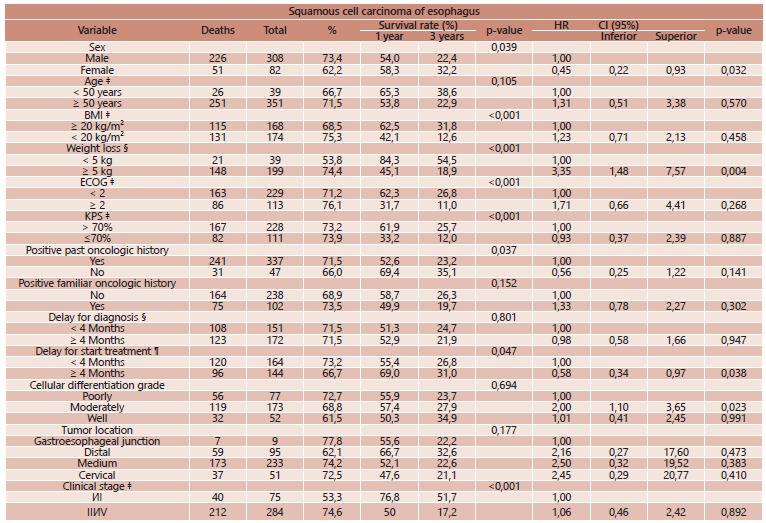
Univariate and multivariate prognostic factors analysis for squamous cell carcinoma

‡=at the time of diagnosis; §=time between initial symptoms to diagnosis; ¶=time between diagnosis and initial oncologic treatment; HR=hazard ratio; ECOG=Eastern Cooperative Oncology Group performance status; KPS= Karnofsky performance status

## DISCUSSION

Several factors have been related to prognosis in esophageal carcinoma^1^ ^,^ ^6^ ^,^ ^12^. 

The present study analyzed prognostic factors associated to patients (age, gender, performance status, past oncologic history, family oncologic history, weight loss and body mass index); factors associated to neoplasm (tumor size, tumor location, grade of cellular differentiation, stage of cancer); and factors associated to treatment (quality of lymphadenopathy, curative intent resection).

### Factors associated to patients

Age had association to bad prognosis only in SCC patients. Eloubeidi et al. also attributed elderly to poor survival rate^5^. Previous paper demonstrated also that family history of esophageal cancer can predict bad prognosis^12^. This finding is not in agreement with the present study.

### Factors associated to neoplasm 

High tumor size and high oncologic stage was associated with high mortality in SCC. This is in accordance with previous papers^5^ ^,^ ^10^. This study evinces SCC with poor cellular differentiation leads to a poor oncologic stage at the moment of diagnosis. Tachibana et al.^10^ also demonstrated an association of differentiation grade and prognosis.

### Factors associated to surgery

Although it could not be demonstrated the relationship between survival and number of dissected lymphnodes, other studies showed a great importance of this variable. 

The number of positive lymph nodes (more vs. less than 5 dissected nodes) is related to an increasing risk of mortality (hazard ratio [HR], 1.29; 95% confidence interval [95%CI], 1.06 -1.56) according to Eloubeidi et al.^5^ Rizk et al.^9^ showed that patients with more than four involved lymph nodes have survival similar to that of patients with M1 disease. Consequently, the number of lymph nodes removed would be an independent factor for prognosis. For Peyre et al ^7^, a minimum of 23 regional lymph nodes should be removed. 

In this study, survival improvement after curative intent surgery must be carefully analyzed, once selection for surgery (only not advanced stages) may be a bias.

## CONCLUSION

 Esophageal carcinoma is a poor prognosis disease. In our study, after five years of follow-up, overall survival is next to 20%. Weight loss (kg), BMI variation (kg/m²) and percentage of weight loss are factors that predict worse stage at diagnosis in the squamous cell carcinoma. In adenocarcinoma, these findings were not statistically significant. 
